# Selection of a set of reliable reference genes for quantitative real-time PCR in normal equine skin and in equine sarcoids

**DOI:** 10.1186/1472-6750-6-24

**Published:** 2006-04-27

**Authors:** Lies Bogaert, Mario Van Poucke, Cindy De Baere, Luc Peelman, Frank Gasthuys, Ann Martens

**Affiliations:** 1 Department of Surgery and Anaesthesiology of Domestic Animals, Faculty of Veterinary Medicine, Ghent University - UGent, Salisburylaan 133, B-9820 Merelbeke, Belgium; 2 Department of Animal Nutrition, Genetics, Breeding and Ethology, Faculty of Veterinary Medicine, Ghent University - UGent, Heidestraat 19, B-9820 Merelbeke, Belgium

## Abstract

**Background:**

Real-time quantitative PCR can be a very powerful and accurate technique to examine gene transcription patterns in different biological conditions. One of the critical steps in comparing transcription profiles is accurate normalisation. In most of the studies published on real-time PCR in horses, normalisation occurred against only one reference gene, usually *GAPDH *or *ACTB*, without validation of its expression stability. This might result in unreliable conclusions, because it has been demonstrated that the expression levels of so called "housekeeping genes" may vary considerably in different tissues, cell types or disease stages, particularly in clinical samples associated with malignant disease. The goal of this study was to establish a reliable set of reference genes for studies concerning normal equine skin and equine sarcoids, which are the most common skin tumour in horses.

**Results:**

In the present study the gene transcription levels of 6 commonly used reference genes (*ACTB, B2M, HPRT1, UBB, TUBA1 *and *RPL32*) were determined in normal equine skin and in equine sarcoids. After applying the geNorm applet to this set of genes, *TUBA1, ACTB *and *UBB *were found to be most stable in normal skin and *B2M, ACTB *and *UBB *in equine sarcoids.

**Conclusion:**

Based on these results, *TUBA1, ACTB *and *UBB*, respectively *B2M, ACTB *and *UBB *can be proposed as reference gene panels for accurate normalisation of quantitative data for normal equine skin, respectively equine sarcoids. When normal skin and equine sarcoids are compared, the use of the geometric mean of *UBB, ACTB *and *B2M *can be recommended as a reliable and accurate normalisation factor.

## Background

Gene expression analysis has become increasingly important in biological research where e.g. gene expression profiles from tissues associated with diseases and disorders have to be compared with each other and with those from normal tissues. One of the most powerful tools in this area is real-time quantitative reverse transcription PCR (qRT-PCR). To account for differences in starting material, RNA preparation, RNA quality and cDNA synthesis, adequate normalisation is frequently performed by comparing expression profiles of the genes of interest with those of constitutively expressed genes (= reference genes). Housekeeping genes are most widely used as reference genes, based on the assumption that they are constitutively expressed in most tissues and under certain circumstances, and that they are more or less resistant to cell cycle fluctuations [1,2]. However, it has been demonstrated that the expression levels of these genes may vary considerably in different tissues, different cell types and different disease stages, particularly in clinical samples associated with malignant disease [3,4]. Housekeeping genes are not only involved in the basal cell metabolism, but appear to participate in other functions too, and therefore are prone to regulation [5-7]. Especially in tumours, the metabolism is generally elevated because of permanent proliferation and expansion. Moreover, some housekeeping genes may have a specific function essential for the tumour metabolism and therefore be up or down regulated [8]. Because of these findings, Vandesompele *et al *[9] proposed to identify a set of stable housekeeping genes in the tissue of interest and use them as internal reference genes for accurate normalisation.

Up till now, only a few gene expression studies using real-time qRT-PCR have been performed in horses. *GAPDH *or *ACTB *were commonly used as a single non-validated reference gene [10-15], since at that time not much information was available concerning this issue. Recently, Waguespack *et al *[16] compared 4 housekeeping genes (*ACTB, B2M, GAPDH *and *TBP*) in the lamellae of the hoof in horses.

In this study, 6 commonly used reference genes in both human and animal studies were investigated, both in normal skin and in equine sarcoids of horses. Equine sarcoids are fibroblastic skin tumours and are the most common tumours in horses. The disease not only induces esthetical defects, but also diminishes the economical value of affected horses [17]. Moreover affected horses show a genetic predisposition for the development of equine sarcoids, through which the breeding value of an animal with sarcoids sharply declines [18-20]. The bovine papillomavirus (BPV) plays an important role in the aetiology of equine sarcoids [21-23]. Several clinical types exist, ranging from small, stable patches to large, aggressive and fast growing tumours [24]. To be able to examine the gene expression profile of BPV in these different clinical types and to compare equine sarcoids with normal skin asymptomatically infected with BPV, a well suited internal control should first of all be established.

## Results and discussion

### Transcription profiling of the candidate genes

cDNA was synthesised from DNA-free RNA (checked with minus RT control) isolated from 8 normal equine skin and 8 equine sarcoid samples. A real-time PCR assay, based on SYBR^® ^Green detection, was designed for the transcription profiling of six frequently used reference genes (*ACTB, B2M, HPRT1, UBB, TUBA1 *and *RPL32 *)in these cDNA samples. During optimisation of the protocol, real-time PCR products were visualised by gelelectrophoresis and sequenced for verification. For every assay, a single amplicon with the expected size was generated without primer dimer formation. Indeed, the formation of primer dimers and unspecific amplification, which can falsely increase the gene expression levels, is a major point of attention, particularly when using intercalating dyes such as SYBR^® ^Green.

Amplicon sequences of *ACTB, B2M, HPRT1*, and *TUBA1 *were 100% identical with the described sequences on which primer design was based. The sequencing of *UBB *revealed 1 gap and 3 SNPs compared to the original sequence (98% identity). The gap is probably due to sequencing errors; the SNPs did not result in an amino acid variation. When translated to amino acid sequence, the *RPL32 *sequence was 100% identical to publicly accessible horse ESTs and human *RPL32 *amino acid sequences.

After optimisation, gene-specific amplification was confirmed by a single peak in melt-curve analysis. For each assay, a standard curve was generated by using 10-fold serial dilutions of pooled cDNA, generated of both normal skin and equine sarcoid tissue, characterised by a linear correlation coefficient (R^2^) varying from 0.991 to 0.998 and a PCR efficiency between 88.1 and 104.6%. These findings showed that these assays are suitable for quantitative purposes.

In order to select a reliable set of reference genes, each assay was performed in duplicate and included the appropriate control samples. To compare the transcription level of the selected genes across the different samples, the Ct values, ranging from 16.4 to 31.4 were converted into raw data based on the PCR efficiency, gathered by standard curve analyses.

### GeNorm analysis

**Figure 1:**
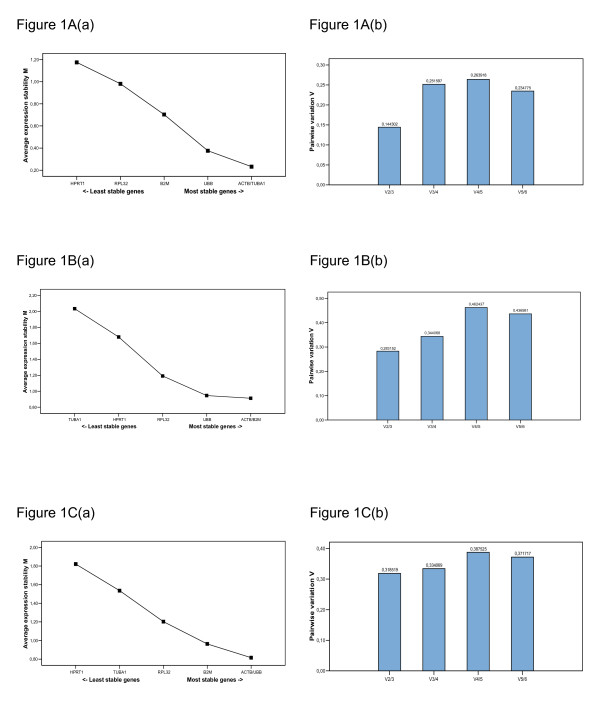
**Gene expression stability of the 6 candidate reference genes analysed by the geNorm program [9]**. (a) Average expression stability values (M) of the control genes, plotted from least stable (left) to most stable (right). (b) Pairwise variation analysis between the normalisation factors NF_n _and NF_n+1_, to determine the optimal number of control genes for normalisation. The higher V_3/4 _value is due to the inclusion of a relative unstable gene and is in accordance with the average expression stability M. A – Normal skin. B – Equine sarcoids. C – Combination of normal skin and equine sarcoids.

The gene expression stability over the different samples was analysed using the geNorm software [9]. The ranking of the 6 candidate reference genes according to their M value was not equivalent between the normal skin samples, the equine sarcoid samples and the combination of both kinds of samples. For normal skin *ACTB, TUBA1 *and *UBB *were the 3 most stable genes (Figure 1A(a)). In equine sarcoids on the other hand, *ACTB, B2M *and *UBB *proved to be the most stable genes with *TUBA1 *being the least stable gene (Figure 1B(a)). When both sets of samples were analysed together, the results showed that the combination of 3 genes (*ACTB, UBB *and *B2M*) is sufficient for adequate normalisation (Figure 1C(a)). The results are listed in Table 1. In another study [16], where *ACTB, B2M, GAPDH *and *TBP *were compared as reference genes, *ACTB *and *B2M *were found to be the best endogenous control genes for real-time qPCR of lamella in the hoof of horses.

When calculating a normalisation factor (NF), a careful choice of the number of reference genes should be made. The more genes included, the more accurate the NF is. However, including too many genes may increase the risk of using unsuitable genes, and is also impractical. On the other hand, if the cut off is made too stringent, stably expressed reference genes may be excluded and accuracy might drop. In order to determine how many reference genes should be included, normalisation factors (NF_n_), based on the geometric mean of the expression levels of the n best reference genes, were calculated by inclusion of an extra, less stable, reference gene according to Vandesompele *et al *[9]. Figures 1A(b), 1B(b) and 1C(b) show the pairwise variation V_n_/V_n+1 _between 2 sequential normalisation factors NF_n _and NF_n+1 _for normal skin, equine sarcoids and the combination of both kinds of samples. In all 3 cases, the inclusion of a 4^th ^gene had no significant contribution (low V_3/4 _value) to the NF. The 3 member sets as described above are a good choice for the calculation of the NF.

### Implementation of results in clinical research

For the reasons discussed above, we have confidence that our gene expression results are accurate and reliable. The described set of reference genes can be used in gene expression studies both in normal skin, equine sarcoids and the combination of both. One of the points of interest in veterinary medicine is the expression level of BPV in different kinds of equine sarcoids. With the normalisation technique described in this study, reliable results can be obtained. Another research topic in this domain is the study of BPV expression in normal skin, showing latent infection with BPV. Also, the expression level of specific horse genes with a putative role in tumourigenesis, can be investigated.

## Conclusion

In conclusion, a method for genomic DNA-free RNA extraction from normal equine skin and equine sarcoids was optimised and a reference gene assay for reliable normalisation of real-time qPCR data, obtained from normal skin, equine sarcoids and the combination of both, was designed. The profiling of the gene expression pattern of 6 putative reference genes showed that 3 reference genes should be used. *ACTB, TUBA1 *and *UBB *can be used in normal skin, while *ACTB, B2M *and *UBB *are the best choice in equine sarcoids. If normal skin and equine sarcoids have to be compared, the same member set as proposed for equine sarcoids can be used.

## Methods

### Sample collection

Eight equine sarcoid samples were obtained from surgically treated horses. A whole range of tumours were sampled (one occult type, one verrucous type, one nodular type and five fibroblastic types). Three sarcoids were located on the medial part of the thigh, two in the axilla and two on the ventral side of the abdomen. Care was taken to obtain only tumoural tissue, without underlying normal stroma. Normal skin samples were obtained from healthy horses undergoing elective surgery or euthanasia (umbilical hernia, castration, osteochondrosis dissecans, dorsal displacement of the soft palatinum). Six of the samples were collected from the ventral abdomen, one from the throat region and one from the shoulder. All samples were freshly collected and stored immediately in RNAlater (Ambion). After overnight incubation at +4°C, the samples were frozen at -18°C until RNA extraction.

### RNA extraction and cDNA synthesis

Total RNA was isolated from the samples using TRIR (ABgene) according to the manufacturer's instructions. Subsequently, approximately 4 μg of the total RNA solution, measured with the BioPhotometer (Eppendorf), were treated with 3 units DNase I (Ambion) to remove genomic DNA. This was followed by a spin-column purification (Microcon YM-10, Millipore). A minus RT control with primers for *GAPDH *was performed to check for successful removal of all the contaminating DNA. These primers were designed by the Primer 3 software [25] using publicly available sequences from the Nucleotide Sequence Database from the National Center for Biotechnology Information (NCBI) [26]. The initial denaturation was performed at 94°C for 10 minutes. Thirty-five cycles of amplification were performed. Each cycle involved a denaturation step of 30 seconds at 94°C, followed by 30 seconds primer annealing at 61°C and 60 seconds primer extension at 72°C. After the last cycle, the PCR-mix was heated during 10 minutes at 72°C to become extension of the partially elongated primers.

First strand cDNA synthesis was carried out on approximately 2.6 μg of the total RNA solution with Superscript™ II Reverse Transcriptase (Invitrogen), an engineered version of M-MLV RT, and a combination of random primers (Invitrogen) and oligo(dT)20 primers (Invitrogen) in a total volume of 20 μl, following the manufacturer's instructions. After this step, a PCR was performed with primers for *GAPDH *to check for the presence of cDNA. The reaction conditions used in this PCR were identical to the PCR described for the minus RT control.

### Reference gene selection and primer design

Six reference genes (*ACTB, B2M, HPRT1, UBB, TUBA1 *and *RPL32*) belonging to different functional classes were selected to reduce the chance that these genes might be co-regulated (Table 2).

The primers, based on horse RNA and DNA sequences found in the NCBI database [26], were designed by the Primer 3 software [25]. The specificity of the primers was tested using a BLAST analysis against the genomic NCBI database. The complete nucleotide sequences of the genes of interest were characterised using Mfold [27] to take into account possible secondary structures at the primer binding sites which might influence the PCR efficiency. The PCR products were cloned (pCR 2.1 vector, Invitrogen) and sequenced for verification (Thermo Sequenase Primer Cycle Sequencing Kit, Amersham Bioscience) with an ALF Express sequencer (Amersham Bioscience). Technical information about the primers and amplicons are listed in Table 3.

### Real-time quantitative PCR

Eight normal skin samples and eight equine sarcoid samples were used for quantification of reference genes.

PCR reactions were performed in a 15 μl reaction volume on the iCycler iQ Real-Time PCR Detection System (Bio-Rad) using the Platinum^® ^SYBR^® ^Green qPCR SuperMix UDG (Invitrogen) supplemented with 0.02 μM fluorescein and 20 ng of cDNA. Primer concentration varied according to the primers used. A blank was incorporated in each assay.

First, an UDG-treatment was done at 50°C to prevent cross contamination. The initial denaturation was performed at 95°C for 2 minutes to activate the *Taq *DNA polymerase, followed by 40 cycles of denaturation at 95°C for 20 seconds and a combined primer annealing/extension at the specific annealing temperature (Table 3) for 40 seconds during which fluorescence was measured. A melt curve was generated to confirm a single gene-specific peak and to detect primer dimer formation by heating the samples from 70 to 95°C in 0.5°C increments with a dwell time at each temperature of 10 seconds while continuously monitoring the fluorescence.

PCR efficiencies were calculated using a relative standard curve derived from a pooled cDNA mixture (a ten-fold dilution series with four measuring points). The pooled cDNA was obtained from normal equine skin and equine sarcoids, using the same RNA isolation and cDNA synthesis protocols as described above.

Each reaction was run in duplicate, whereby a no-template control was included.

During optimisation of the protocol, the PCR products were loaded on a 3% agarose gel after each run to confirm specific gene amplification and the absence of primer dimer formation.

### Determination of reference gene expression stability

To determine the stability of the selected reference genes, the geNorm Visual Basic application for Microsoft Excel was used as described by Vandesompele *et al *[9]. This approach relies on the principle that the expression ratio of two perfect reference genes should be identical in all samples, independent of the experimental condition or cell type.

## Authors' contributions

LB was the primary author of the manuscript, was responsible for the primer design and determined the study design. MVP participated in the study design and provided real-time support. CDB performed most of the experimental procedures. LP, AM and FG participated in the design of the project, helped to draft the manuscript and supervised the study. All authors read and approved the final manuscript.

## Figures and Tables

**Table 1:** Ranking of the reference genes. The reference genes are ranked in order of their expression stability in normal equine skin, in equine sarcoids and in the combination of both kinds of samples, decreasing from top to bottom. The reference genes chosen to calculate the normalisation factor, used for comparing equine sarcoids and normal skin, are printed in bold.

***Normal skin***	***Equine sarcoid***	***Combination***
*TUBA1*	***B2M***	***UBB***
***ACTB***	***ACTB***	***ACTB***
***UBB***	***UBB***	***B2M***
***B2M***	*RPL32*	*RPL32*
*RPL32*	*HPRT1*	*TUBA1*
*HPRT1*	*TUBA1*	*HPRT1*

**Table 2:** Name and function of genes mentioned in the text

Symbol*	Gene name*	***Function***
*ACTB*	actin, beta	Cytoskeletal structural protein
*B2M*	beta-2-microglobulin	Beta-chain of major histocompatibility complex class I molecules
*HPRT1*	hypoxanthine phosphoribosyltransferase 1	Purine synthesis in salvage pathway
*UBB*	ubiquitin B	Protein degradation
*TUBA1*	tubulin, alpha 1 (testis specific)	Structural protein
*RPL32*	ribosomal protein L32	Member of the 80 different ribosome proteins
*GAPDH*	glyceraldehyde-3-phosphate dehydrogenase	Glycolytic enzyme
*TBP*	TATA box binding protein	General RNA polymerase II transcription factor

* Nomenclature according to Genew [28]

**Table 3:** Information on the primers used in this study

***Gene***	***Genbank accession number***	***Primer sequence***	***Ta (°C)***	***Product size (bp)***	***Primer concentration***
*ACTB*	AF035774	CCAGCACGATGAAGATCAAG GTGGACAATGAGGCCAGAAT	59	88	0.5 μl 10 μM
*B2M*	X69083	GTTCCATCCGCCTGGAGATT GGGGGTCTTTGAGAGTAGAGTG	60	182	0.25 μl 10 μM
*HPRT1*	AY372182	GGCAAAACAATGCAAACCTT CAAGGGCATATCCTACGACAA	60	163	0.5 μl 10 μM
*UBB*	AF506969	GCAAGACCATCACCCTGGA CTAACAGCCACCCCTGAGAC	61	206	0.5 μl 10 μM
*TUBA1*	AW260995	GCCCTACAACTCCATCCTGA ATGGCTTCATTGTCCACCA	60	78	0.5 μl 10 μM
*RPL32*	CX594263	AGCCATCTACTCGGCGTCA TCCAATGCCTCTGGGTTTC	61	149	0.25 μl 10 μM
*GAPDH*	AF157626	GATGCCCCAATGTTTGTGA AAGCAGGGATGATGTTCTGG	61	250	0.5 μl 10 μM
